# Harnessing machine learning to find synergistic combinations for FDA-approved cancer drugs

**DOI:** 10.1038/s41598-024-52814-w

**Published:** 2024-01-29

**Authors:** Tarek Abd El-Hafeez, Mahmoud Y. Shams, Yaseen A. M. M. Elshaier, Heba Mamdouh Farghaly, Aboul Ella Hassanien

**Affiliations:** 1 Department of Computer Science, Faculty of Science, Minia University, El-Minia, Egypt; 2 Computer Science Unit, Deraya University, El-Minia, Egypt; 3 Faculty of Artificial Intelligence, Kafrelsheikh University, Kafr El-Sheikh, Egypt; 4 Faculty of Computers and Artificial Intelligence, Cairo University, Cairo, Egypt; 5 Department of Organic and Medicinal Chemistry, Faculty of Pharmacy, University of Sadat City, Sadat City, Menoufia Egypt; 6 Scientific Research Group in Egypt (SRGE), Cairo, Egypt

**Keywords:** Cancer, Drug discovery, Molecular biology, Stem cells, Diseases, Health care, Computer science, Information technology, Scientific data

## Abstract

Combination therapy is a fundamental strategy in cancer chemotherapy. It involves administering two or more anti-cancer agents to increase efficacy and overcome multidrug resistance compared to monotherapy. However, drug combinations can exhibit synergy, additivity, or antagonism. This study presents a machine learning framework to classify and predict cancer drug combinations. The framework utilizes several key steps including data collection and annotation from the O’Neil drug interaction dataset, data preprocessing, stratified splitting into training and test sets, construction and evaluation of classification models to categorize combinations as synergistic, additive, or antagonistic, application of regression models to predict combination sensitivity scores for enhanced predictions compared to prior work, and the last step is examination of drug features and mechanisms of action to understand synergy behaviors for optimal combinations. The models identified combination pairs most likely to synergize against different cancers. Kinase inhibitors combined with mTOR inhibitors, DNA damage-inducing drugs or HDAC inhibitors showed benefit, particularly for ovarian, melanoma, prostate, lung and colorectal carcinomas. Analysis highlighted Gemcitabine, MK-8776 and AZD1775 as frequently synergizing across cancer types. This machine learning framework provides a valuable approach to uncover more effective multi-drug regimens.

## Introduction

As research into cancer cell abnormalities continues, an increasing number of anti-cancer medications are being developed and assessed*.* However, the efficacy of one medication or a single target drug as a monotherapy is limited due to innate or acquired resistance. To address this challenge, a more effective approach is drug combination treatment. The use of drug combinations has proven to be an effective strategy for treating diseases that are challenging to manage, including cancer and infectious infections^1–4^. The use of drug combination therapy can inhibit multiple targets, thereby overcoming drug resistance in infectious fungal diseases^5–7^. The explanation for this is that biological systems are less capable of correcting for the action of two or more medications at the same time^2,6,8,9^. Effective medication combinations have traditionally been established by evaluating all potential combinations of a pre-defined set of pharmaceuticals in an experimental setting^6,10^. The screening of drug combinations is a challenging task due to the vast number of available drugs, which makes the process time-consuming, labor-intensive, and expensive. With n medications, there are n(n−1)/2 possible pairwise drug combinations, as well as multiple higher-order combinations. Furthermore, the constant development of new pharmaceuticals results in an exponential growth in the number of potential pharmacological combinations that can be tested each year^10^. As a small number of compounds can yield a large number of combinations^7^ testing all possible pharmacological combinations would be a resource-intensive and time-consuming endeavor. Even with high-throughput screening, conducting limited drug combination trials would only scratch the surface of the vast number of potential drug combinations. Therefore, utilizing experimental screening as a means of identifying optimal medication combinations remains a challenging task^11,12^. Thus, it is not easy to identify optimal drug combinations using the experimental screen approach. There is a significant need to develop tools that can identify optimal drug pairs for more effective and synergistic cancer treatment. Recent technological advancements have ushered in a new era of precision medicine that combines machine learning (ML) and biomedical science to provide data-driven assessments of diseases. Leveraging machine-learning algorithms is a powerful tool within the broader field of artificial intelligence. It can extract meaningful conclusions by leveraging big data, making it an increasingly popular tool for cancer detection and treatment. The ultimate goal of precision medicine is to provide therapies that not only increase the chances of patient survival but also improve their quality of life by reducing unwanted side effects. This can be achieved by matching patients with appropriate therapies or therapeutic combinations. The main objective of this paper is to utilize a machine learning technology to predict effective drug synergy pairs for cancer treatment. The proposed approach involves several steps, including data collection and annotation, data preprocessing, partitioning the dataset into training and test sets, building classification and regression models, testing, and validating the most suitable models. Additionally, it examines the drug features and mechanisms of action to better understand the synergy behavior of the best combination therapy. The key contributions of this paper include annotating each drug combination by its generic name and mechanism of action. The ML is adapted to classify synergism, additive, and antagonism class labels, determining the best combination of CSS score among the same cancer cell line, classifying data by the type of cancer tissue based on six cancer types, and identifying the synergistic drug combinations for each cell line. This paper significantly advances cancer chemotherapy research by developing a machine learning to classify and predict effective drug combinations.

The main contributions of this paper are summarized as follows:

- We enhanced predictive modeling through ML-based classification/regression for identifying synergistic, additive, and antagonistic drug pairs.
- Integrating regression with classification models to quantify drug interactions that provides deeper insights compared to previous and related work.
- We leveraged the comprehensive O'Neil drug interaction dataset that ensuring robustness across cancer types and relying on curated data strengthens our findings.
- The proposed model successfully identified combination pairs that shows the highest likelihood of synergy against specific cancers. Notably, coupling kinase inhibitors with mTOR, DNA damage or HDAC inhibitors showed promise, highlighting strategies for effective multi-drug regimens.
- We examined drug features and mechanisms to understand why certain pairs synergize which focusing on consistently effective drugs like gemcitabine, MK-8776, and AZD1775 against various cancers added context.

Finally, systematically uncovering advantageous multi-drug options has potential to inform clinical decision making. The highlighted regimens for ovarian, melanoma, prostate, lung, and colorectal cancers could significantly impact personalized cancer therapy development.

Therefore, the proposed innovative machine learning-based drug combination model for classifying, predicting, and rationalizing synergistic drug combinations makes an important advance. We believe the findings offer potential to enhance combination therapy effectiveness and ultimately improve patient outcomes.

The structure of the rest of this paper is organized as follows. Section 2 reviews related work. Section 3 introduces preliminary concepts. Section 4 details the methodology and the proposed model. Sections 5–6 present experimental results and discussion. Section 7 discusses the conclusion and future work.

## Related work

Synergistic effects between drugs, being rare and highly context-dependent, necessitated the development of novel approaches for patient stratification in optimal therapy regimens, especially in personalized combinatorial treatments. Computational methods played a crucial role in systematically screening combination effects in-silico, prioritizing potent combinations for further testing amid the vast number of potential options. A systematic literature review presented by Kong et al.^13^ encompassing 117 computational methods that classified these methods based on their combination prediction tasks and input data requirements to aid researchers in selecting appropriate prediction methods for diverse real-world applications. While most methods focused on predicting or classifying combination synergy, few considered the efficacy and potential toxicity, key determinants of therapeutic success. There is a pressing need for the development of methods enabling dose-specific predictions across multiple doses, essential for clinical translation and model-based identification of biomarkers predictive of heterogeneous drug combination responses. Despite the prevalent focus on anticancer applications in the reviewed methods, many modeling approaches are applicable to antiviral and other diseases or indications.

Cancer remains the leading cause of death globally, and the economic and financial burden of cancer research is increasing. Chemotherapy for cancer relies on using two or more therapeutic drugs in combination. By adopting a synergistic or additive approach, combining anti-cancer medications enhances the efficacy of a monotherapy strategy. This technique can mitigate drug resistance, minimize the cytotoxicity of administered drugs, and improve the survival rate of cancer patients^14^. Developing a novel anti-cancer medicine is expensive and time-consuming, involving in vitro and in vivo investigations and clinical trials before being approved by the FDA. In^14–16^, a newly developed medicine takes around 15 years to reach the pharmaceutical market. Therefore, in^17^, Combination treatment is being studied because it gives efficient and effective results at a low cost. Pharmaceuticals exert their action by interacting with specific cell components called receptors or active sites, which are dictated by the drug's chemical and physical properties. In a drug-drug combination, each medication interacts with its receptor or the same receptor. Prior research has primarily focused on defining synergy, quantitatively calculating dose–effect curves, and determining whether a specific drug combination can achieve a synergistic effect based on established synergy criteria and experimental findings. Since Loewe devised the Loewe additive model in 1926 to characterize synergistic drug combinations, other academics have worked on drug combination studies^18–25^. Loewe^20^ developed the Loewe additive equation to evaluate whether a certain medicine combination would synergistically impact. Chou and Talalay^25–27^ proposed the median-effect called CI-Isobologram index as well as the dose-reduction index formula^25^ for drug-drug interactions. In their approach, C_I1_, = 1, and > 1 imply synergism, additive effect, and antagonism, respectively. Greco also created the universal response surface technique, a novel way to assess drug interactions (URSA)^28^. However, a few models can be used to predict whether or not a certain drug combination would have a synergistic impact. Some methods for reducing the number of drug combination experiments have been developed in recent years. Jansen et al.^19^ identified potential combinatorial drugs using chemogenomic profiles. First, they looked at data from sensitivity-based chemogenomic profiles found in the literature and profiling trials. Then, any drug pair with chemogenomic profiles similar to the known synergy pairings was deemed an antifungal synergy candidate. Chen et al.^29^ used a combination of fractional factorial design and stepwise regression to dramatically reduce the time required to uncover synergistic drug combinations.

On the other hand, both of these tactics rely heavily on the results of biological research. Li et al.^30^ created the topological and agent score parameters to analyze the synergistic connection for certain medication combinations. They created the NIMS algorithm to discover potential synergistic medication combinations on a wide scale. By combining molecular and pharmacological data, Zhao et al.^31^ represented pharmaceuticals using a set of attributes and created a revolutionary computational approach for prioritizing prospective medication combinations. Huang et al.^32^ used clinical side-effect data and the drug label to predict drug combinations. Three FDA-black-boxed major side effects were shown to have the greatest impact on prediction accuracy. Furthermore, they developed DrugComboRanker, a computational method for prioritizing synergistic medication combinations based on the development and segmentation of functional drug networks. Yin et al.^33^ showed that pharmacological synergy or antagonism is a property of target-related network topology and investigated various basic synergistic and antagonistic patterns, implying that designing novel synergistic drug combinations based on network topology might be beneficial. To construct a sparsity-induced classifier for potential synergistic drug combination inference, Iwata et al.^34^ used drug-target interactions, drug anatomical therapeutic chemical categorization system codes, and known synergistic drug combinations from the Orange Book and KEGG DRUG databases. Chen et al.^35^ developed a unique network-based synergistic medication combination prediction model based on systematic pathway–pathway interactions. However, only computational models have been built, and none of the above research has found any experimental validation.

The current efforts for drug combinations are performed. Sun et al.^36^ demonstrate a ranking system of Anti-Cancer Synergy (RACS) that integrates features of targeting networks and transcriptome profiles and validates it on three cancer types.

Even though the molecular mechanism driving specific interactions is unknown, RACS has the potential to greatly enhance drug synergy prediction and minimize experimental prescreening of current pharmaceuticals for repurposing to cancer treatment.

Li et al.^37^ used synergistic drug combinations to find new ways to treat cancer. However, precise prediction of synergistic drug combinations is challenging due to the unknown mechanisms of pharmaceutical synergism. A variety of factors, including drug response and target networks, can aid in predicting synergistic medicine combinations. The influence of drug chemical structural characteristics, drug target network features, and pharmacogenomics features on discovering synergistic medication combinations were investigated.

Xia et al.^38^ used the National Cancer Institute's (NCI-60) drug pair screening program against 60 well-characterized human tumor cell lines offers a unique resource for modeling combinational drug action. They provide a computer model for predicting cell line response to selecting drug combinations in the NCI-ALMANAC database. They show that pharmacological descriptions have the most predictive value, and that deep learning can predict combinational drug responses with promising results.

Malyutina et al.^39^ stated that many computational methods only examine the synergy of drug combinations, not their sensitivity, leading to misleading positive results. They developed a novel cross design to medication combination sensitivity and synergy testing more cost-effective and simultaneous. They created a medication Combination Sensitivity Score (CSS) to measure a drug pair's sensitivity. They demonstrated that the CSS is highly reproducible between replicates, indicating that it can be used as a reliable metric. They also demonstrated that CSS could be predicted using machine learning techniques that used top pharmacy features to cluster cancer cell lines based on their medication combination sensitivity profiles.

Jiang et al.^40^ proposed a Graph Convolutional Network (GCN) model for predicting synergistic medication combinations in specific cancer cell lines. The GCN technique addressed a link prediction difficulty using a convolutional neural network model to conduct heterogeneous graph embedding. They looked at the most widely predicted pharmaceutical combinations in various cancer cell lines. They discovered that many had been proven to have synergistic anti-cancer action in vitro or in vivo against the same or different tumors. The findings imply that this study might be used to in silico identify and enhance synergistic drug combinations.

Liu and Xie^41^ reduced medication resistance while increasing therapeutic efficacy. Several new synergistic medicine combinations have been predicted with high confidence for ovarian cancer, with few treatment options. Because of the growing number of anti-cancer drugs, assessing all therapeutic combinations is both costly and time-consuming. To address these problems, they developed TranSynergy as a knowledge-enabled and self-attention transformer augmented deep learning model that enhances medicine combination prediction performance and interpretability. This might aid researchers in the discovery of novel anti-cancer drugs and biomarkers for precision medicine. Table 1 briefly describes the current efforts for drug combination strategies.

**Table 1:** The comparative study of the current drug combination strategies and the applied dataset.

Author/year	Dataset used	Algorithm	Problem statement	Major contribution	Pros	Recommendation
Sun et al.^36^	DCDB 2.0 drug combination database^42^	Semi-supervised learning	It exhibits an Anti-Cancer Synergy Ranking System (RACS)	They got a probability concordance of 0.78	Synergy prediction and significantly minimize experimental prescreening of current pharmaceuticals for repurposing to cancer treatment	One prediction is confirmed in vivo in a zebrafish MCF7 xenograft model, suggesting great synergy and minimal toxicity. The approach was validated using A549 lung cancer cells
Li et al.^37^	Dream^43^	Random Forest	The goal of synergistic drug combinations is to find new ways to treat cancer	Drug chemical structure, drug target network, and pharmacogenomics features all have an effect	Three of the 28 anti-cancer drug combinations identified by the prediction algorithm were effective	The use of a prediction model could assist in narrowing the search area and speed up the discovery of clinically effective synergistic medication combinations
Xia et al.^44^	NCI-ALMANAC^45^	Deep Learning	Predicting the response of a selection of medication combinations in cell lines	Predicting tumor progression. While they achieve the greatest results using a combination of molecular feature types	Based on the model's expected combination, they rate the medicine combos for each cell line	They demonstrate promising results in predicting combinational medication response using deep learning
Malyutina et al.^39^	O'Neil^46^	Elastic Net, Random Forest, Support Vector Machine	Combinations in cancer have been aided by high-throughput drug screening	Cross-design to medication combination sensitivity and synergy testing	S synergy score was created by comparing the dose–response of a drug combination to a single drug dose–response	Overall, they proved the effectiveness of combining cross-design with CSS sensitivity and S synergy rating
Jiang et al.^40^	O’Neil^46^	Graph Convolutional Network	A combination of various networks to predict synergistic medication combinations	The GCN model for predicting synergistic medication combinations in specific cancer cell lines in this study. The GCN technique	Using a large heterogonous network, the GCN model was able to correctly predict cell line-specific synergistic drug combinations	Predicting and optimizing synergistic medication pairings in silico
Liu et al.^41^	O’Neil^46^	Transformer boosted Deep Learning	Drug combinations have shown considerable promise in the treatment of cancer	An improved deep learning model for drug combination prediction that enhances performance and interpretability. Through gene–gene interaction, cell-line gene reliance, and genome-wide drug-target interaction	In comprehensive benchmark testing, TranSynergy beats the state-of-the-art approach	Identify biomarkers for precision medicine. For ovarian cancer, which has few therapy choices

Chen et al.^47^ explored the role of small molecules, low-weight organic compounds, in influencing diseases by inhibiting specific protein functions or disrupting protein–protein interactions. Focused on microRNAs (miRNAs) as crucial elements in cellular biology with potential as diagnostic and therapeutic targets. The review highlighted successful screenings of drug-like compounds against various miRNAs, demonstrating the feasibility of targeting miRNAs with small molecules. Covered five aspects of miRNA functions, summarized disease states linked to miRNA alterations, and introduced small molecules associated with key miRNAs. The study also discussed publicly accessible databases and web servers related to small molecule-miRNA associations, emphasizing their importance in biomedical research. Reviewed experimental techniques and computational models for identifying small molecule-miRNA associations, along with a discussion of limitations and future directions for computational model development. The urgent need for effective drugs to address complex human diseases led to a reevaluation of drug discovery strategies. Traditional approaches, which were time-consuming and costly, adhered to the one drug-one target paradigm. However, recent studies indicated that drugs typically influenced related pathways rather than single targets, prompting the introduction of a new strategy called pathway-based drug discovery. The review presented by Wang et al.^48^ outlined the importance of identifying associations between drugs and pathways They introduced the background of drugs and the concept of drug-pathway associations, listed publicly accessible databases and web servers, and categorized state-of-the-art computational methods into Bayesian spare factor-based, matrix decomposition-based, and other machine learning methods.

### Consent statement

This article does not contain any studies with human participants or animals performed by any of the authors.

## Preliminaries

This work employs classification techniques to assign a class to an unseen record properly. Furthermore, the Naive Bayes (NB), Random Forests (RF), K-Nearest Neighbor (KNN), and Logistic Regression (LR) classifiers are used to accurately find actual synergistic, additive, and antagonistic medication combinations. The mechanisms of action for these medication combinations are categorized into two groups using synergy scores within the ranges of [− 5, 5] and [− 10, 10] which yields the most accurate findings, helping identify the actual synergistic, additive, and antagonistic medication combinations.

### Machine learning models

#### Classification models

##### Naïve Bayes (NB) model

NB^49,50^ is a widely used method for classification and is particularly suitable when the input dimensionality is high. Despite its simplicity, NB can often outperform more complex classification techniques. It measures the probability of each input feature (attribute) for a predictable state. The Bayesian classifier uses the Bayes rule to calculate the posterior probability for each class c_i_. NB is based on the simplifying assumption that the features, y, are independent of the class. Therefore, the probability can be calculated by using the conditional probabilities of each feature given in the class. So, the posterior probability, *P (C*_*i*_*|y),* is expressed as in Eqs. (1) and (2).

1 $$ P(C_{i} |y)\, = \,P(y|C_{i} )P(C_{i} ) / P(y). $$

where

2 $$ {\text{P}}\left( y \right) = \mathop \sum \limits_{i = 1}^{n} \left( {{\text{y}}|C_{i} } \right){\text{C}}_{i} $$

where n is the number of classes such that:

- P (C_i_): A priori likelihood of class C_i_.
- P(y): the likelihood density for feature y.
- P (y|C_i_): the class-conditional likelihood density of the feature y that belongs to the C_i_ class.
- P (C_i_|y): the posterior probability of the C_i_ class when observing y.

##### Random forests (RF) model

The RF^51^ is defined as an ensemble learning method for classification and regression. Ensemble learning techniques (such as boosting, bagging, and RF) have great interest since they are robust to noise and more accurate than single classifiers. RF is a collection of tree structure classifiers. Each tree is trained with a subset of the training data that are randomly selected (i.e. bootstrapped), with the same distribution of samples for all the trees in the forest. The final classification is then built based on the majority of trees in the forest. In other words, RF tries to build several decision trees with initial variables and various data samples and then combine predictions to make the final decision. For an RF that consists of N trees, the prediction of the class label c of case x by majority voting is made using Eq. (3).

3 $$ l(x) = argmax_{c} \left( {\mathop \sum \limits_{n = 1}^{N} {\text{I}}_{{{\text{h}}_{{\text{n}}} }} { }\left( {\text{X}} \right) = {\text{c}}} \right). $$

where h_n_ is the nth tree of the RF, and I is the indicator function.

##### Logistic Regression (LR) Model

The LR^52^ is a linear model used for classification problems. LR measures the relationship between the response (dependent) variable and one or more explanatory (independent) variables for a given dataset that indicates the significance and strength of the impact of the explanatory variables on the response variable. The response variable is a class label that we are trying to predict. However, the explanatory variables are the features or attributes used to predict the class label. The output of LR is the probability that given input points belong to a certain class. Typically, LR estimates probabilities using the logistic function, also known as the sigmoid function, which is given in Eq. (4).

4 $$ f(y) \, = \frac{l}{{1{ } + {\text{ e}}^{{ - {\text{k}}\left( {{\text{y}} - {\text{y}}_{0} } \right)}} }}. $$

where e denotes the natural logarithm base, L denotes the curve's maximum value, y_0_ denotes the sigmoid midpoint's y value, and k denotes the curve's logistic growth rate or steepness.

##### K-nearest neighbor (KNN) model

The KNN classifier is an instance-based non-parametric classifier^53^. This approach is based on estimating the nearest neighbor. The new instances are categorized using a distance metric to measure similarity. The *K* in KNN stands for the number of nearest neighbors' data items. The main concept of the KNN model is that a new instance's prediction is formed by scanning the whole training set for comparable K neighbor examples and classifying them according to the class with the most occurrences. To discover a comparable situation, the Euclidean distance formula is employed. As shown in Eq. 5, Euclidean distance is equal to the square root of the sum of squared differences between the new instance (A_i_) and the current instance (B_j_)^54^.

5 $$ Euclidean_{i,} = \sqrt {\mathop \sum \limits_{k = 1}^{n} \left( {{\text{A}}_{{\text{ik }}} - {\text{ B}}_{{{\text{jk}}}} { }} \right)^{2} } $$

#### Regression models

We provide state-of-the-art machine learning algorithms for forecasting the sensitivity of a medication combination based on the massive quantity of drug combination data gathered in the O'Neil dataset. We investigated three basic machine-learning prediction techniques: linear regression, random forest regression, and ridge regression.

##### Linear regression model

Regression models are statistical models for estimating or forecasting the target or dependent variable using independent variables. Linear regression^55^ is a regression model that estimate or forecast the target or dependent variable using independent variables.

Equation (6) shows the relationship between dependent and independent variables. Each univariate analysis in the linear regression model is used to how much the dependent variable will predict each independent variable.

6 $${\text{Y}}={\upbeta }_{0} + {\upbeta }_{1}{{\text{X}}}_{1} + {\upbeta }_{2}{{\text{X}}}_{2} + \cdots {\upbeta }_{{\text{p}}}{{\text{X}}}_{{\text{p}}} +\upepsilon $$

where $Y$ is the total number of new cases and *X*1, *X*2,…, and *Xp* are p independent. $\upbeta 0$, $\upbeta 1$, $\upbeta 2$, …, *and* $\mathrm{\beta p}$ are the intercept and coefficients of the variables, respectively. $\upepsilon$ is the error term in the model.

##### Random forest regression model

Random forest regression^51^ has become a popular technique in a variety of prediction scenarios^39,41,56^ due to its high accuracy and ability to handle a large number of features. A regression tree is a nonlinear regression model in which samples are partitioned at each binary tree node depending on the value of a single input variable. By generating a set of regression trees in which the training set for each tree is chosen using Bootstrap sampling from the original sample set. Then, the features considered for partitioning at each node is a random subset of the original set of features. Random forest combines the two concepts of bagging and random feature selection. The random selection of variables assessed for partitioning at each node and the bootstrap sampling for each regression tree creation lower the correlation between the constructed regression trees. It is meaning that averaging their prediction responses will minimize error variance.

##### Ridge regression model

The ridge regression technique was first discussed in 1970^57^. Ridge regression is used to reduce the impact of collinearity in linear regression when the independent variables have a substantial correlation. The regression coefficients in the generic regression model are described in Eq. (7).

7 $${\text{b}}={\left({X}^{T }X\right)}^{-1} {X}^{T } Y$$

where b is the coefficients vector, X denotes the (n*p) data matrix with p independent variables (each with n observations), X^T^ signifies the transpose of X, and Y denotes the (n*1) matrix containing the regression's dependent variable.

## The proposed synergistic combinations for FDA-approved cancer drugs model

**Figure 1:**
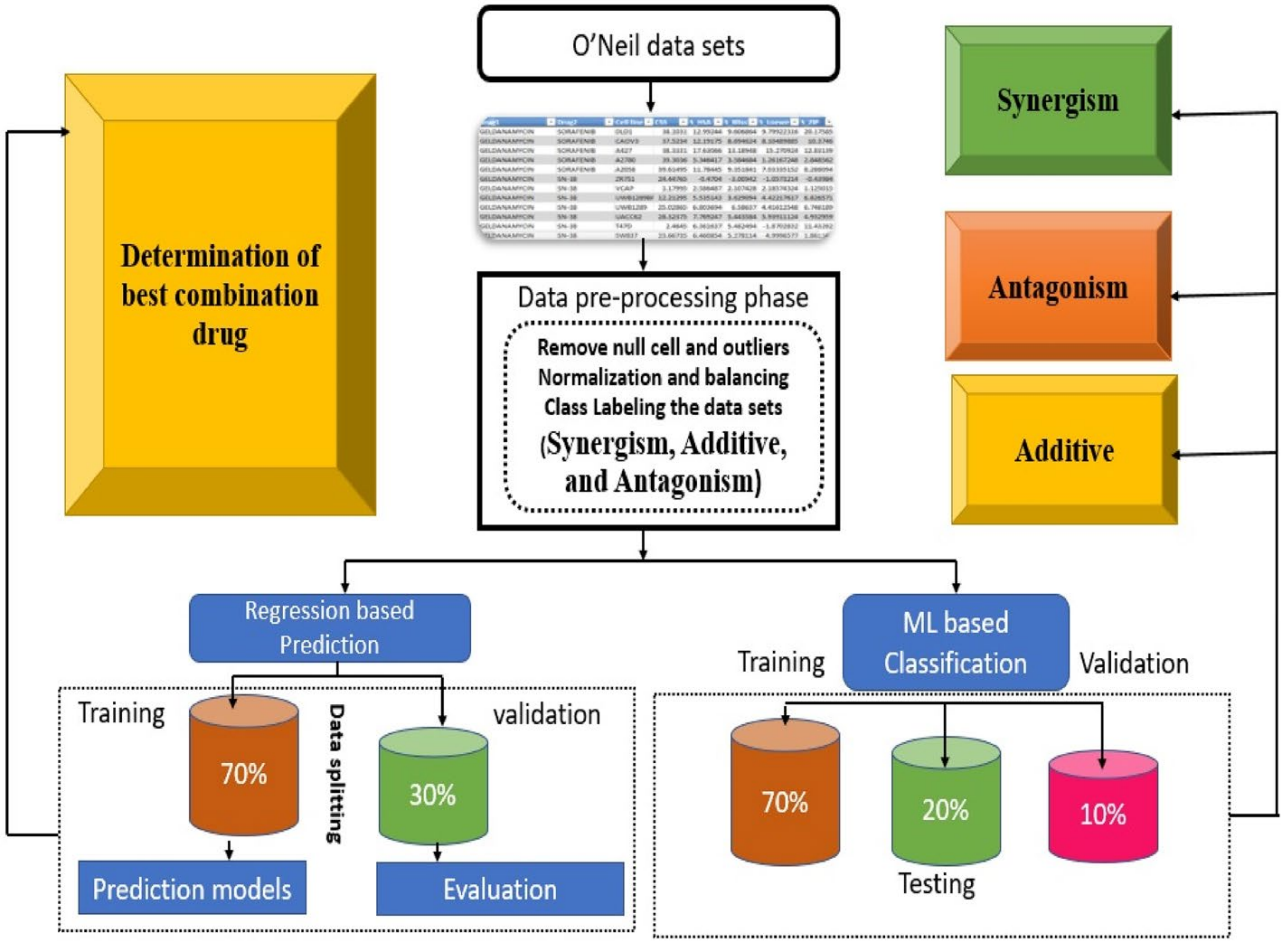
The general architecture model of the proposed drug combination.

Drug combinations are of great interest for cancer treatment. We designed a machine-learning framework to identify effective drug synergy pairs out of all possible combinations. Figure 1 investigates the general structure of the proposed model for drug combination, which includes three main parts. Part (1) is the preprocessing of the enrolled data. Part (2) is the classification of the combined drugs in terms of Synergism, Antagonism, and Additive. Part (3) demonstrates the prediction of the best combination drugs.

The proposed model is utilizing for predicting the best combination of drugs based on O’Neil data sets. It uses a combination of classification and regression models. The classification part takes pre-processed data and classifies the interaction between two drugs as synergism, antagonism, or additive. This is done using a training set that makes up 70% of the data, and then validated on a 20% validation set. The remaining 10% is used for testing the final model.

The regression part uses the same training and validation sets, but instead of predicting the class (synergism, antagonism, additive.). It predicts a numerical value for the best combination drug.

The classification and regression parts are essential for the proposed model. The classification part helps to identify promising drug combinations. By knowing whether two drugs are likely to have a synergistic, antagonistic, or additive effect, as we focus on the most promising combinations. While the regression part provides a more precise estimate of the interaction effect. This can be used to rank different drug combinations and select the most effective ones.

**Figure 2:**
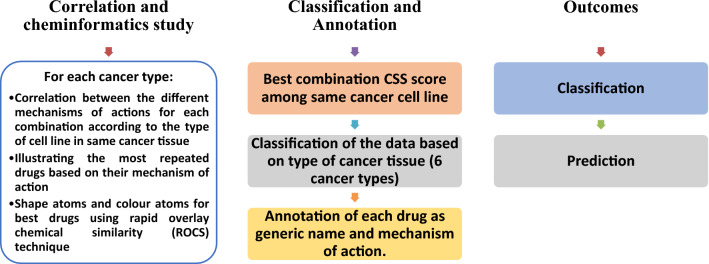
Analysis of generated results based on drug mechanism of action and type of cancer tissue.

The analysis of the two major tasks of the current drug combination study, including both classification and prediction outcomes shown in Fig. 2. We investigate the prediction steps to determine the sensitivity score for a drug combination model in Fig. 1. We utilized the O'Neil dataset and annotated the enrolled data to classify each drug combination's synergism, additive, and antagonism. The three-class labels use different synergy scores from [− 5 to 5] and [− 10 to 10] intervals. The Figure investigated the classification and annotation to determine the best combination score CSS among the same cancer cell line. In the following subsection, detailed descriptions of the utilized dataset are investigated.

### Drug dataset characteristics

The data set illustrated the effect of drug combination against different cell lines with calculations of synergy scores as follows:

- *Drug 1* generic name of the first drug
- *Drug 2* generic name of the second drug
- *Cell line* cancer cell lines
- Synergy scores
  - *S-HSA* is the single drug's maximum impact,
  - *S-Bliss* is the predicted result of two drugs acting independently,
  - *S-Loewe* is the predicted result of a drug combined with itself,
  - *S-ZIP* is the predicted interaction between two drugs that do not potentiate each other,
  - *CSS* is a drug combination sensitivity score (CSS) used to determine the sensitivity of a drug pair.

#### The O’Neil drug combination data

The drug combination sensitivity (CSS) grading was used for the O'Neil medication combination data^39,58^, which contains 22,737 medication combinations including 38 different treatments in 39 cancer cell lines covering seven different tissue types. The O'Neil data is thought to be of good quality^46^, as it contains multiple replicates and has been utilized in previous machine learning development^39,59–61^ In the first phase, single-drug screening was performed using six replicates and eight concentrations to calculate the IC50 value for each medication. In the second stage, a four-by-four dose matrix was utilized to cover the range of IC50 concentrations for a drug pair with four replicates, utilizing a four-by-four dose matrix. To employ the cross design, just the row and column corresponding to the concentrations closest to the IC50 of the individual drugs were chosen. The CSS1 and CSS2 values were shown to be closely associated (Pearson correlation = 0.82) when the IC50 concentrations of each drug were utilized. The CSS1 and CSS2 values varied from 0 to 50, with a 5.62 difference in absolute value. A CSS score may be interpreted right away as a normalized average percent inhibition of the pharmaceutical combination response, as illustrated in Eq. (8).

8 $$CSS=\frac{\left(CSS1+CSS2\right)}{2}$$

The CSS1 and CSS2 correlations are generated to assess the CSS values' stability further. The correlations are close to zero (Pearson correlation = 0.075), showing that the substantial association is attributable to CSS's resiliency in real-world pharmaceutical combinations. There was a strong association between the CSS value and the values produced from individual replicates (minimum Pearson correlation = 0.97). Because the CSS1 and CSS2 values in the medicine combination sensitivity score are often constant, averaging them as a summary for the medicine combination sensitivity score is recommended.

**Figure 3:**
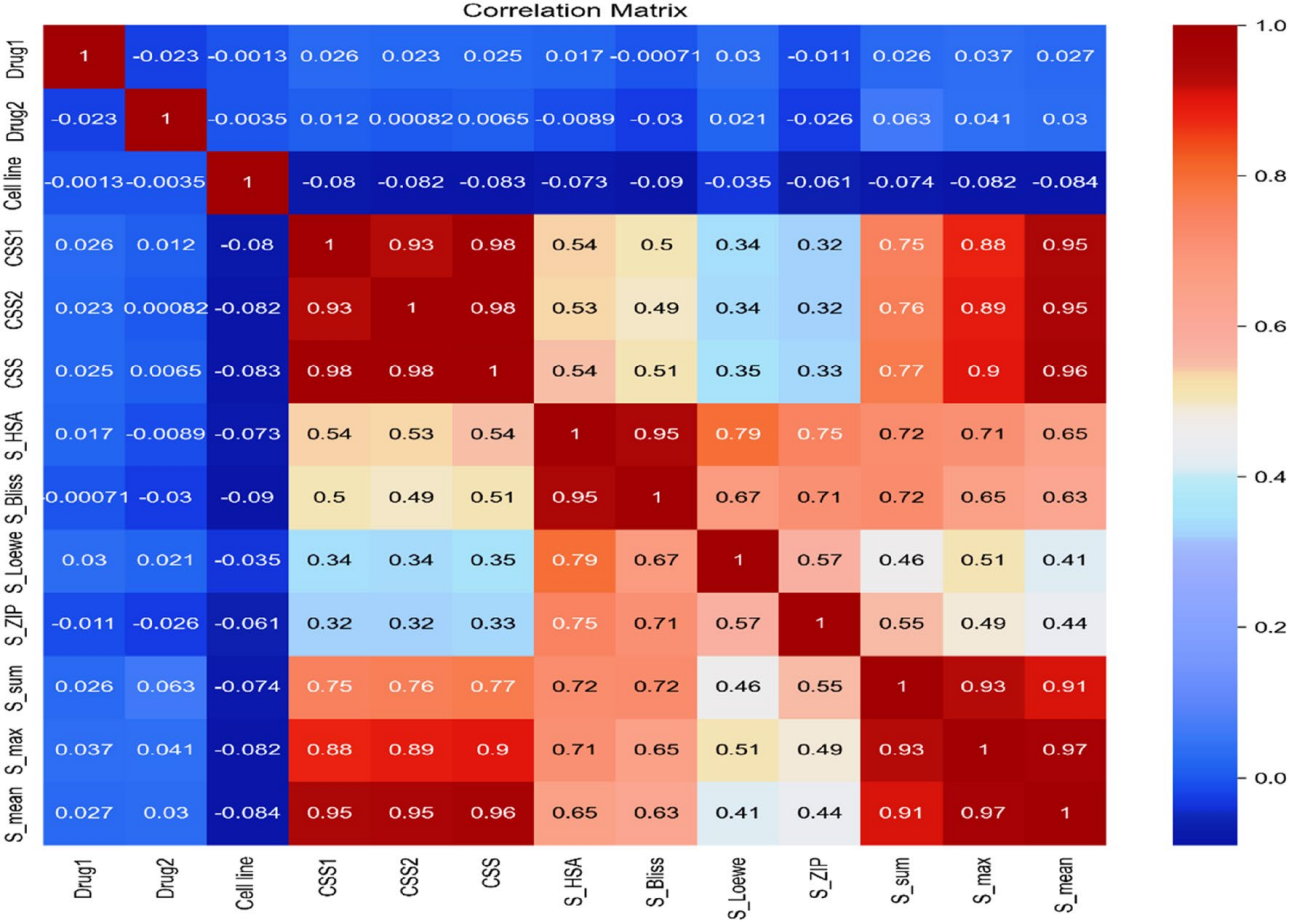
The correlation heat map of the proposed drug combination model.

The O'Neil dataset's numerical variable correlation is shown in Fig. 3. Each row and column in the correlation matrix represents a continuous variable, and each value indicates the correlation coefficient (Pearson’s R-value) between the variables represented by that row and column. Most attributes are highly correlated, according to our observations.

Table 2 presents the Pearson correlation coefficients between different variables in the dataset. The Pearson correlation coefficient measures the linear relationship between two variables, ranging from − 1 to + 1. A positive correlation coefficient indicates a positive linear relationship, while a negative correlation coefficient indicates a negative linear relationship.

**Table 2:** Pearson correlation.

Row #	Correlation	Second feature	First feature
1	+ 0.983	CSS	CSS2
2	+ 0.982	CSS	CSS1
3	+ 0.967	S_max	S_mean
4	+ 0.965	CSS	S_mean
5	+ 0.950	CSS2	S_mean
6	+ 0.945	CSS1	S_mean
7	+ 0.931	CSS1	CSS2
8	+ 0.930	S_max	S_sum
9	+ 0.910	S_mean	S_sum
10	+ 0.901	CSS	S_max
11	+ 0.890	CSS2	S_max
12	+ 0.879	CSS1	S_max
13	+ 0.768	CSS	S_sum
14	+ 0.760	CSS2	S_sum
15	+ 0.749	CSS1	S_sum

From Table 2, we observe several strong positive correlations between various variables. For instance, in rows 1 and 2, the CSS and CSS2 have a correlation coefficient of + 0.983 and + 0.982, respectively, indicating a very strong positive linear relationship between these two variables. Similarly, in rows 3 and 4, the S_max and S_mean exhibit a correlation coefficient of + 0.967 and + 0.965, respectively, suggesting a strong positive linear relationship between these variables.

Furthermore, rows 5 and 6 show a correlation coefficient of + 0.950 and + 0.945, respectively, indicating a relatively strong positive linear relationship between CSS2 and S_mean, as well as between CSS1 and S_mean.

Other notable correlations include the positive relationships between CSS1 and CSS2 in row 7 with a correlation coefficient of + 0.931, and between S_max and S_sum in row 8 with a correlation coefficient of + 0.930.

On the other hand, some correlations are relatively weaker such as the correlation coefficients between CSS, S_max, CSS2, and S_sum in rows 10, 11, 12, 13, 14, and 15, ranging from + 0.901 to + 0.749.

Since our models require existing drug combinations for training, we retrieve the mechanism of action of these drugs in combination therapy using synergy scores. The synergy scores can be interpreted as the average excess response due to drug interactions. There is no threshold to define a good synergy score. Therefore, we made annotation for the dataset according to four synergy scores determined for each drug combination as follow:

First, we are using a synergy score of [− 5, 5] range as follows^39^:

- *Less than* − 5 indicates that the interaction between two drugs will most likely be antagonistic.
- *Between* − 5 *and* 5 the interaction between two drugs is most likely additive.
- *If the number is more than 5* the interaction between two drugs is likely to be synergistic.

Second, we are using a synergy score of [− 10, 10] range as follows^59^:

- Less than − 10: indicates that the interaction between two drugs will most likely be antagonistic.
- From − 10 to 10: the interaction between two drugs is likely to be additive.
- *Larger than *10 the interaction between two drugs is likely to be synergistic.

### Data preprocessing phase

Data preprocessing is the first step in the proposed system to identify and process some attributes’ noisy, incomplete, irreverent, and inconsistent values. For the O'Neil dataset, data cleaning is performed by removing any missing values. Moreover, many outliers need to be handled properly, and the dataset is not properly distributed.

Outlier classification is a critical issue in machine learning because certain data samples may have considerably different features than others in the same class, and so get isolated from the rest of the data in that class. As a result, we used the Interquartile Range (IQR) technique to find outliers in this study^62^. IQR can eliminate outliers by dividing a rank-ordered sample into four equal halves, known as quartiles, and evaluating dispersion. Q1 and Q3 denote the middle value in the first and second halves of the rank-ordered dataset, respectively, while Q2 is the median value for the whole set. Q3 minus Q1 gives the IQR. Outliers are data points that fall outside the Q1 1.5 IQR or the Q3 + 1.5 IQR.

A random under-sampling algorithm ensures that the data is more evenly distributed and does not cause bias. The O'Neil dataset has an unbalanced class distribution. The random under-sampling algorithm can be applied to the dataset to overcome this. Using a random under-sampling algorithm, all of the data points from the minority class are used. Instances are randomly removed from the majority training set until the desired balance is achieved.

Because raw data has such a broad range of values, a normalization approach (also known as feature scaling) is used to adjust the values of numeric columns in the dataset to achieve a common scale, allowing the related objective functions to function effectively^63^. In this study, we use the min–max normalization technique.

### Machine learning classifiers phase

Classification approaches are used to accurately detect actual synergistic, additive, and antagonistic medication combinations with great accuracy. The mechanism of action of these pharmacological combinations is categorized into two categories based on synergy scores: a [− 5, 5] range and a [− 10, 10] range, as previously noted. The classifiers Naive Bayes (NB), Random Forests (RF), K Nearest Neighbor (KNN), and Logistic Regression (LR) are used to evaluate which synergy scores range delivers correct results. There were two techniques employed. Without balancing the data and eliminating outliers, one can use the data straight to machine learning algorithms. The results obtained were not encouraging. As a result, we eliminate outliers and balance data before examining the impact of data preparation on classification model performance.

### Regression phase

We use state-of-the-art machine learning methods to estimate the sensitivity of a medicine combination based on the vast volume of drug combination data compiled in the O'Neil dataset. We looked at three important machine-learning methods for predictions: linear regression, random forest regression, and ridge regression. After analyzing the results, we then identify the synergy score that is more correlated to the prediction of the CSS score for the drug combination mechanism, identify the synergistic drug combinations for each cell line, and determine the best CSS score range for each drug combination mechanism.

### Model evaluation metrices

The quality of the models was gauged based on well-known evaluation metrics such as the accuracy of the classification, precision, recall, and F1-scores for classification and the Mean Absolute Error (MAE), R squared score(R^2^), and Mean Squared Error (MSE) for regression.

#### Evaluation metrics for classification models

Equations (9), (10), (11), and (12) are determined by the confusion matrix performance that represents the accuracy, precision, recall, and F1-score, respectively.

9 $${\text{Accuracy}}=\frac{\mathrm{TP }+\mathrm{ TN}}{\mathrm{TP }+\mathrm{ FP }+\mathrm{ TN }+\mathrm{ FN}}$$

10 $${\text{Precision}}=\frac{\mathrm{TP }}{\mathrm{TP }+\mathrm{ FP}}$$

11 $${\text{Recall}}=\frac{\mathrm{TP }}{\mathrm{TP }+\mathrm{ FN}}$$

12 $${\text{F}}1 -\mathrm{ score }=2* \frac{\left(\mathrm{Precision }\times \mathrm{ Recall}\right)}{\left(\mathrm{Precision }+\mathrm{ Recall}\right)}$$

These metrics are based on a “confusion matrix” that includes true positives (TP), true negatives (TN), false positives (FP), and false negatives (FN)^64^.

#### Evaluation metrics for regression models

The determination coefficient R-square is one of the most common performances used to evaluate the regression model as shown in Eq. (13). On the other hand, the Minimum Acceptable Error (MAE) is shown in Eq. (14), while the Mean Square Error (MSE) is investigated in Eq. (15).

13 $${{\text{R}}}^{2}=\frac{\sum {\left(y-\dot{\widehat{y}}\right)}^{2}}{\sum {\left(y-\dot{\overline{y}}\right)}^{2}}$$

14 $${\text{MAE}}=\frac{\sum_{i=1}^{n}\left|\widehat{{y}_{i}}-y\right|}{{\text{n}}}$$

15 $${\text{MSE}}=\frac{\sum_{i=1}^{n}\left|\widehat{{y}_{i}}-{y}_{i}\right|}{{\text{n}}}$$

where y is the actual value, $\dot{\widehat{{\text{y}}}}$ is the corresponding predicted value, $\dot{\overline{{\text{y}}}}$ is the mean of the actual values in the set, and *n* is the total number of test objects^65^.

## Experimental results and analysis

In this section, we have conducted experiments to assess the performance of the machine learning framework for identifying effective synergetic drug combinations. As mentioned before, the O'Neil drug combination dataset is used for machine learning framework construction. We are conducting our experiments on a 3 GHz i5 computer with a 4 GB main memory and 64-bit Windows 10 operating system. The experiment is carried out using the Python programming language.

Initially, the focus of the first part of this section is on using classification techniques to correctly detect the mechanisms of action of the drug combinations that are synergistic, additive, and antagonistic with high accuracy. Then check the influence of data preprocessing on the performance of the classification models. In the second part, we focus on applying regression models to predict the sensitivity of a drug combination. Then identify the synergy score that correlate to the prediction of the CSS score for the drug combination mechanism, identify the synergistic drug combinations for each cell line, and determine the best CSS score range for each drug combination mechanism. Finally, we illustrated the mechanism of action for each drug and the name of the cancer type. Then, drug features were calculated based on the Rapid Overlay Chemical Similarity (ROCS) analysis technique.

### Building classification and regression models

Many classification and regression models were created utilizing various machine-learning approaches to find successful synergistic medication combinations.

#### Identifying the mode of action of the drug combinations

Drug combinations were generally overlooked in terms of effectiveness, synergy, and mechanisms of action. As a result, we attempt to categorize medications as synergistic, additive, or antagonistic drugs using machine kinds in this investigation. Well-known classification approaches are used to identify actual synergistic, additive, and antagonistic medication combinations with high accuracy. Synergy scores of [− 5, 5] and [− 10, 10] are used to classify the mechanisms of action of certain medication combinations. Then the evaluation metrics listed in Sec. 4.5.1 are used to measure the performance of classification techniques and determine which synergy scores range gives accurate results. The default parameters for each classification technique were used. The experiments were done by using a 10 cross-validation method. Table 3 and Table 4 show the performance results for all classifiers to classify drugs according to the mechanism types using two ranges of synergy scores [− 10, 10] and [− 5, 5], respectively.

**Table 3:** Comparison of different classifiers using a synergy score of [− 10, 10] range.

Method	Accuracy	Precision	Recall	F1-score
NB	96.196	73.308	97.575	81.765
RF	**99.927**	**99.676**	**98.633**	**98.571**
KNN	98.715	93.224	93.004	92.633
LR	92.548	73.559	93.675	79.771

Significant values are in [bold].

**Table 4:** Comparison of different classifiers using a synergy score [− 5, 5] range.

Method	Accuracy	Precision	Recall	F1-score
NB	92.537	77.874	89.727	82.157
RF	**99.916**	**99.693**	**98.785**	**99.600**
KNN	96.758	95.000	92.001	93.225
LR	91.085	84.426	82.161	81.785

Significant values are in [bold].

**Figure 4:**
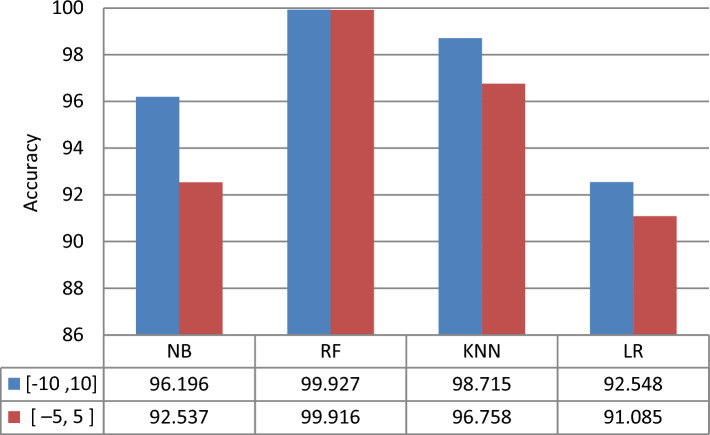
Comparative results of classification techniques using two synergy score ranges in terms of accuracy.

Tables 3 and 4 show that the performance of classification techniques when using a synergy score of [− 10, 10] range to identify the mechanism types of the drugs consistently produced the best accuracy. Figure 4 shows comparative results of classification techniques using two synergy score ranges in accuracy. When utilizing the synergy score [− 10, 10] range to apply classification approaches, it can be shown that RF provided the best accuracy of the four classifiers evaluated, while LR produced the worst. It's worth noting, however, that even the worst-performing LR model outperformed a synergy score in the [− 5, 5] range in terms of accuracy. Therefore, in this study, using the synergy score [− 10, 10] range was the best option for identifying the mechanism types of the drugs, as shown in Fig. 4.

#### Influence of data preprocessing on the performance of the classification models

The performance machine learning model depends significantly on the quality of the data and the strategy of using the data^66^. Therefore, the assessment of the influence of data preprocessing on machine-learning models' performance has a high significance. We first removed the null values from the O’Neil dataset to optimize the classifier performance. Then we analyzed the entire data distribution to check the class distribution. The impacts of outliers and data imbalance on classification performance were then investigated. As we inferred, the synergy score [− 10, 10] range was the best option for identifying the mechanism types of the drugs.

**Figure 5:**
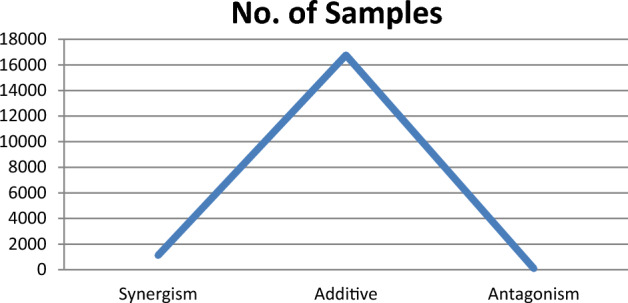
The class distribution of the three classes Synergism, Additive, and Antagonism.

*Checking the distribution of the data* The data distribution plays an important role when the prediction or classification of a problem is to be done. After removing the null values, the O’Neil dataset has 1119 synergisms, 16764 additives, and 99 antagonisms out of all drug combinations as shown in Fig. 5. Therefore, we need to balance the dataset, or otherwise, it might get overfit.

#### Removing outliers and data balancing.

One of the most difficult problems for machine learning classifiers is learning from outliers and unbalanced data. The O'Neil dataset has two flaws: it is imbalanced and has outliers, as we discovered. We handle these issues: Using the IQR technique, we first eliminated the outliers. We used the random under-sampling approach to get a balanced dataset in the second stage. Table 5 shows the results after removing outliers and balancing the dataset when using a synergy score of [− 10, 10] range.

**Table 5:** Performance comparison of different classifiers after applying data preprocessing techniques.

Method	Accuracy	Precision	Recall	F1-score
Naïve	98.80	96.667	96.11	96.363
RF	98.80	95.00	91.281	86.266
KNN	99.60	95.0	94.80	94.897
LR	94.80	86.167	83.998	84.948

From Table 5, it is evident that when the data preprocessing method is applied, it can improve the performance of classifiers in terms of F1 scores, precision.

Recall and accuracy. These results also showed that the KNN classifier had the best performance while LR produced the least. Moreover, we noticed that the NB, KNN, and LR trained with preprocessed data produce better precision than when trained with original data.

According to these findings, the RF also performed poorly in all performance criteria and did not respond well to the applied selective data preparation process.

**Figure 6:**
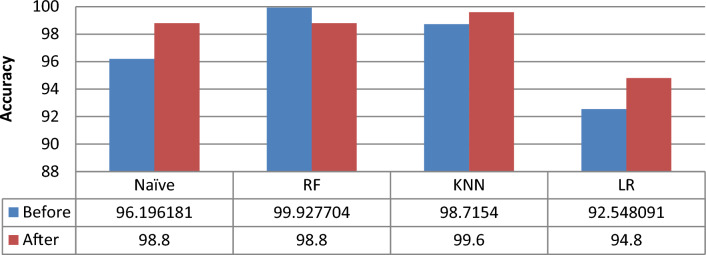
Comparison of different classifiers with and without applying data preprocessing techniques.

Figure 6 compares the performance of machine learning models with a synergy score in the [− 10, 10] range before and after eliminating outliers and balancing data in terms of accuracy. It can be observed that, in most cases, classification algorithms perform better when they are trained with preprocessed data.

#### Predicting the drug combination sensitivity score (CSS) using regression model

We wanted to see how accurate various machine-learning approaches were in predicting the CSS for each drug combination after discovering the CSS. Linear regressions, random forest regressions, and ridge regressions were examined as three state-of-the-art machine-learning algorithms for CSS prediction. We randomly chose 70% of the medication combinations to train different machine-learning models. The performance of the regression model is then measured using the evaluation metrics specified in Sect. 4.5.2. for the remaining 30% of the unique medication combinations utilized as testing data, the model with the lowest MSE and MAE was chosen to predict the CSS values.

**Figure 7:**
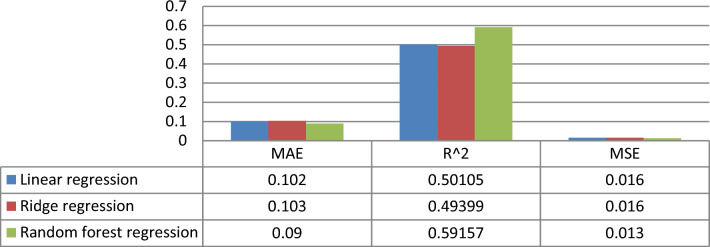
Performance comparison of different prediction methods in terms of MAE, R^2^, and MSE.

Figure 7 shows the comparative results of prediction methods to predict the CSS score of the drug combination. We observed that the random forest regression model achieved the best performance to predict the CSS, with an MAE of 0.09 and MSE of 0.013.

To determine the synergy score that is more correlated to the prediction of the CSS score for each class, we use Pearson’s correlation method. Table 6 shows the Pearson correlations of the S synergy scores with the CSS score for each drug combination mechanism. We investigated that the most feature correlated to CSS prediction is the HSA score for the additive and synergistic drug combinations, whereas the Loewe score for the antagonistic drug combinations.

**Table 6:** Pearson’s correlation of the S synergy scores with the CSS Score for each drug combination mechanism.

	S_HSA	S_Bliss	S_Loewe	S_ZIP
Synergism	**0.369212**	0.282605	0.278984	− 0.005884
Additive	**0.287297**	0.266139	0.113107	0.015478
Antagonism	0.525654	0.496790	**0.535932**	0.234947

Significant values are in [bold].

#### Statistical analysis

An Analysis of Variance (ANOVA)^67,68^ was conducted to investigate the presence of statistically significant differences among different machine-learning algorithms. ANOVA is a parametric statistical test suitable for comparing means across different groups. It evaluates the variation in a dataset to determine if it is attributable to genuine differences in group means or if it could occur by chance. The null hypothesis posits that there is no significant difference in the population means of the groups, while the alternative hypothesis suggests unequal means. The chosen significance level was 0.05. The results are summarized in Table 7.

**Table 7:** ANOVA test results for the machine learning models.

	Sum of squares	*Df*	Mean Square	F-statistics	*P* value
Between groups	221.502	3	73.834	4.900	.019
Within groups	180.819	12	15.068		
Total	402.321	15			

As shown in Table 6, the F-statistic is 4.900, and the associated *p* value is 0.019. Since the *p* value is less than the conventional significance level of 0.05, there is evidence to reject the null hypothesis, suggesting that there are significant differences among the group means.

#### Comprehensive insights into the key hyperparameters of the regression models

Tables 8 and 9, present a comprehensive insight into the key hyperparameters governing the behavior of regression models and classifiers employed in our analysis. Understanding these hyperparameters is fundamental to optimizing the performance and interpretability of machine learning models.

**Table 8:** Overview of hyperparameters for regression models.

Model	Hyperparameter	Description	Value
LinearRegression	No hyperparameters specified in the code		
Lasso	alpha	Regularization parameter	1.0
RandomForestRegressor	n_jobs	Number of parallel jobs (− 1 for all CPUs)	− 1
RandomForestRegressor	random_state	Random seed for reproducibility	42
BayesianRidge	No hyperparameters specified in the code		
DecisionTreeRegressor	max_depth	Maximum depth of the decision tree	3
Ridge	No hyperparameters specified in the code		
KNeighborsRegressor	n_neighbors	Number of neighbors to consider	5
KNeighborsRegressor	weights	Weighting function for predictions	‘uniform’

**Table 9:** Overview of hyperparameters for classification Models.

Classifier	Hyperparameters	Description	Value
Naive Bayes (Naive)	No hyperparameters	No hyperparameters are specified for Naive Bayes	N/A
Random Forest (RF)	- n_estimators	Number of trees in the forest	100
	- max_depth	Maximum depth of the tree	None (i.e., nodes are expanded until containing less than min_samples_split samples)
	- min_samples_split	Minimum samples required to split a node	2
	- min_samples_leaf	Minimum samples required to be at a leaf node	1
K-Nearest Neighbors (KNN)	- n_neighbors	Number of neighbors to use for classification	5
	- weights	Weight function used in prediction	‘uniform’
	- algorithm	Algorithm used to compute nearest neighbors	‘auto’
Logistic Regression (LR)	- C	Inverse of regularization strength	1.0
	- penalty	Type of regularization	‘l2’

### Comparative analysis

In this section, we perform a comparative study between other recent approaches with the proposed method using the same dataset utilized in this study as shown in Table 10. The SNRMPACDC model presented by Li et al.^69^ introduced a fusion of various neural network components. It employed a Siamese convolutional network to analyze individual drug features, capturing potential interactions between them. Additionally, the model utilized random matrix projection to reduce the dimensionality of drug features while preserving pertinent information. The convolutional network processes cancer cell line features, extracting relevant information. Lastly, a multi-layer perceptron integrates all the processed features and produces a predicted score indicating the synergy of drug combinations. The evaluation of their model is based on both regression and classification prediction.

**Table 10:** The comparative study between recent approaches and the proposed model using the same dataset in this study.

Author	Method	Results	Comment
Li et al.^69^	SNRMPACDC: Siamese CNN, Random Matrix Projection, MLP	RMSE: 15.01, Pearson correlation: 0.75 (regression)	Good regression & classification (AUC 0.91, AUPR 0.62)
Huang et al.^70^	Kaplan–Meier, Cox Regression, Nomogram	AUC > 0.800 for 3-, 5-, and 10-year intervals	Well-calibrated nomogram for clinical use
Zhang et al.^71^	Sequential model with various feature encodings	M13-M20 performance metrics documented	Comprehensive feature analysis, confidence intervals included
Kuru et al.^72^	MatchMaker: Deep learning with drug structure & gene expression	DrugComb: 15% correlation, 33% lower MSE	Deep learning with large dataset and good performance
Zagidullin et al.^73^	Drug and cell line categorization in DrugComb	33.3% of drugs lacked documented mechanisms	Highlighted data limitations in DrugComb
Tang and Gottlieb^74^	Biologically motivated deep learning for pathway features	MSE: 70.6 ± 6.4, topologically interacting pathways for synergy	Considered pathway interactions for improved prediction
El Khili et al., 2023 ^75^	MARSY: Deep learning multitask model for synergy prediction	RMSE: 9.06 (± 0.45) for drug-pair combinations	Efficient prediction for large datasets with multitask learning
Proposed model	Classification (NB, RF, KNN, and LR)	Average Accuracy: 89%	After applying data preprocessing
	Regression (Linear, Ridge, and RF regressors)	Average MAE: 0.0984 R^2^: 0.5290 MSE:0.015	Prediction methods were employed to forecast the CSS score of drug combinations

Firstly, in regression prediction they achieved Root mean-squared error (RMSE) of 15.01 and Pearson correlation coefficient of 0.75. While in classification prediction they achieved Area under the receiver operating characteristic curve (AUC) of 0.91 ± 0.03 and area under the precision-recall curve (AUPR) of 0.62 ± 0.05. Huang et al.^70^ proposed Kaplan–Meier method and univariate Cox regression analysis for predictive accuracy and clinical utility of the nomogram through a calibration curve. They used ROC curve, and decision curve analysis (DCA). The ROC curves for all independent prognostic factors were plotted to confirm the superior predictive validity of the nomogram compared to a single independent prognostic factor. Results from the calibration curve, ROC analysis, and DCA collectively demonstrated the nomogram’s performance and suitability for clinical application, with areas under the ROC curve exceeding 0.800 for 3-, 5-, and 10-year intervals.

Zhang et al.^71^ presented the progression of five models is detailed sequentially, starting with label information that involves categorical encoding of both drugs and cell lines. Subsequent steps include incorporating the chemical structure of drugs encoded by molecular fingerprints and cell line cancer gene expression. Further enhancements involve the addition of monotherapy efficacy, followed by the incorporation of dose–response curve baseline features and imputation features. The performance metrics for all models are documented as M13–M20. The confidence for evaluation metrics, expressed as a 95% confidence interval, is established by employing bootstrapping techniques on predictions derived from the complete datasets.

A model presented by Kuru et al.^72^ used MatchMaker, to predict drug synergy scores by incorporating both drug chemical structure information and cell line gene expression profiles within a deep learning architecture. The model utilized the most extensive drug combination dataset to date, DrugComb. Through MatchMaker their model achieved 15% correlation and 33% lower mean squared error (MSE). Zagidullin et al.^73^ presented a categorization of drugs and cell lines, along with their respective proportions in DrugComb, involved classifying drugs based on mechanism types. Notably, 33.3% of the drugs (n = 756) lacked well-documented mechanisms of action according to major databases.

A biologically motivated deep learning strategy to extract pathway-level features from molecular data of drugs and cell lines presented by Tang and Gottlieb^74^. They aiming to predict drug synergy and quantify interactions in synergistic drug pairs and their approach yielded a mean squared error (MSE) of 70.6 ± 6.4. Additionally, their findings suggest that drug combinations exhibit greater synergy when their top contributing pathways are closely interconnected on a protein interaction network, implying a potential strategy for combination therapy involving topologically interacting pathways. El Khili et al.^75^ introduced a deep-learning multitask model named MARSY (Multitask drug pAiR SynergY), incorporating information from the gene expression profiles of cancer cell lines. MARSY was employed to predict synergy scores for 133,722 drug-pair cell line combinations. The performance evaluation, conducted through a fivefold leave-pair-out approach, resulted in a root mean square error (RMSE) of 9.06 (± 0.45) for MARSY and baseline methods.

#### Determination of the best CSS score range for each drug combination mechanism

Upon the previous experimental results, when filtering out the drug combinations, we found that the true synergistic ones are those with a CSS score consistently higher than 28, true antagonistic ones are those with a CSS score consistently lower than 8, and the true additive drug combinations those with the CSS score consistently between [− 8, 28] range. Each cell line was chosen based on the drug pairings with the greatest predicted CSS scores to identify the synergistic drug combinations for each cell line. For the A2058 cancer cell line, the combination of GEMCITABINE and MK-8776 was the most effective. Table 11 shows the top forecasts for the remaining cell lines.

**Table 11:** Highest drug combination scores with a type of tissue cancer and drug pair information.

Reference that confirms our results	Our results						
	Cell line	Tissue disease	Drug 1		Drug 2		CSS
			Name	Mechanism	Name	Mechanism	
^76^	A2058	Epithelial/ Melanoma	Gemcitabine	CDK inhibitor	MK-8776	Inhibit phosphorylation at ser296-Chk1	42.96965
^77^	A375	Epithelial/ Melanoma	Mitomycin	Alkylating agent, crosslink DNA	BEZ-235	mTOR inhibitor or	42.9484
^40^	HT144	Human skin malignant melanoma	Geldanamycin	Inhibits the function of Hsp90	BEZ-235	mTOR inhibitor	39.05215
^78^	RPMI7951	Human skin malignant melanoma	Etoposide	topoisomerase II inhibitors	Geldanamycin	inhibits the function of Hsp90	43.3456
^79^	SKMEL30	Cutaneous melanoma	Doxorubicin	topoisomerase II inhibitor	MK-8776	Inhibits phosphorylation at ser296-Chk1	41.24685
^40^	UACC62	Human Melanoma	Geldanamycin	inhibits the function of Hsp90	BEZ-235	mTOR inhibitor	39.7059
^80^	OVCAR3	Ovarian cancer	Gemcitabine	CDK inhibitor	MK-8776	Inhibits phosphorylation at ser296-Chk1	42.3738
^81^	A2780	Epithelial/ ovarian cancer	Gemcitabine	CDK inhibitor	Dinaciclib	CDK inhibitor	43.1521
^82,83^	ES2	Ovary carcinoma	BEZ-235	mTOR inhibitor	MK-8776	Inhibits phosphorylation at ser296-Chk1	43.2467
^84^	CaoV3	High-grade ovarian serous adenocarcinoma	Mitomycine	Alkylating agent, crosslink DNA	AZD1775	WEE1 activity and induces DNA damage	43.2587
^85^	PA1	Ovary (Teratocarcinoma)	L778123	inhibitor of FPTase and GGPTase-I	MK-5108	Aurora A kinase inhibitor	43.4017
^86^	OV90	Ovaery Malignant Papillary Serous Adenocarcinoma	AZD1775	WEE1 activity and induces DNA damage	MK-8776	Inhibit phosphorylation at ser296-Chk1	30.11825
^87^	UWB1289	Ovarian carcinoma	ERLOTINIB	an epidermal growth factor receptor inhibitor	Sorafenib	Kinase inhibitor	39.3318
^88^	UWB1289BRCA1	Ovarian carcinoma	Bortezomib	Inhibits the 26S proteasome, preventing the activation of NF-κB	BEZ-235	mTOR inhibitor	40.7658
^89^	SKOV3	Ovarian Adenocarcinoma	AZD1775	WEE1 activity and induces DNA damage	MK-8776	Inhibits phosphorylation at ser296-Chk1	42.1901
^90^	HCT116	Colorectal carcinoma	Gemcitabine	CDK inhibitors	AZD1775	Inhibit WEE1 activity and induce DNA damage	40.6917
^91^	COLO320DM	Colon adenocarcinoma	Methotrexate	DHFR inhibitor	Erlotinib	EGFR inhibitor	41.28255
^80^	DLD1	Adenocarcinoma; Colorectal	Gemcitabine	CDK inhibitor	MK-8776	Inhibit phosphorylation at ser296-Chk1	34.00605
^92^	HT29	Human colon adenocarcinoma cell	AZD1775	Inhibit WEE1 activity and induce DNA damage	MK-8776	Inhibits phosphorylation at ser296-Chk1	41.93325
^93^	LOVO	Adenocarcinoma; Colorectal	AZD1775	Inhibits WEE1 activity and induces DNA damage	MK-8776	Inhibits phosphorylation at ser296-Chk1	42.70565
^94^	RKO	Colon carcinoma	Vinblastine	binds to microtubular proteins in the mitotic spindle	Sorafenib	kinase inhibitor	39.98395
^95^	SW620	Adenocarcinoma of the colon	Bortezomib	Inhibits the 26S proteasome, preventing the activation of NF-κB	BEZ-235	mTOR inhibitor	43.17505
^96^	SW837	Human colorectal adenocarcinoma	Dasatinib	protein tyrosine kinase inhibitor	BEZ-235	mTOR inhibitor	36.07155
^40^	LNCAP	Prostate adenocarcinoma	Bortezomib	Inhibits the 26S proteasome, preventing the activation of NF-κB	BEZ-235	mTOR inhibitor	42.39735
^97^	VCAP	Prostate cancer	Gemcitabine	CDK inhibitor	AZD1775	WEE1 activity and induces DNA damage	40.90855
^98^	OCUBM	Breast carcinoma	Gemcitabine	CDK inhibitor	AZD1775	WEE1 activity and induces DNA damage	42.46835
^82^	T47D	Human breast cancer	MK-8669	mTOR inhibitor	BEZ-235	mTOR inhibitor	34.71975
^83^	KPL1	Human breast cancer	MK-8669	mTOR inhibitor	BEZ-235	mTOR inhibitor	34.43505
^99^	MDAMB436	Human Breast Cancer	Bortezomib	Inhibits the 26S proteasome, preventing the activation of NF-κB	Geldanamycin	Inhibits the function of Hsp90	41.08135
^46^	EFM192B	Breast carcinoma	Mitomycine	Alkylating agent, crosslink DNA	ZOLINZA	HDAC inhibitor	37.10165
^100^	SKMES1	Lung squamous cell carcinoma	Topotecan	Topoisomerase inhibitor	BEZ-235	inhibits mTOR	41.80635
^101,102^	A427	Lung adenocarcinoma	AZD1775	Inhibits WEE1 activity and induces DNA damage	MK-8776	Inhibit phosphorylation at ser296-Chk1	43.352
^69^	MSTO	Lung carcinoma	Vinorelbine	Tubulin inhibitors	Zolinza	HDAC inhibitor	43.3598
^80^	NCIH1650	Lung carcinoma	Gemcitabine	CDK inhibitor	MK-8776	Inhibits phosphorylation at ser296-Chk1	42.68605
^80^	NCIH2122	Non-small cell lung cancer	Gemcitabine	CDK inhibitor	MK-8776	Inhibit phosphorylation at ser296-Chk1	42.00935
^103^	NCIH23	Human lung cancer	Zolinza	HDAC inhibitor	AZD1775	WEE1 activity and induces DNA damage	42.78825
^40^	NCIH460	Large cell cancer of the lung	Geldanamycin	inhibits the function of Hsp90	BEZ-235	mTOR inhibitor	41.1219
^41^	NCIH520	Squamous Cell Carcinoma	MK-8669	mTOR inhibitor	BEZ-235	mTOR inhibitor	41.70995

We noticed that most drugs mentioned in this combination model are with similar mechanism of action which suggests the approach that drug combinations occur for drugs with same pharmacodynamic and different pharmacokinetic profiles.

### Cheminformatics studies

#### Drugs and their mechanism of actions

In drug combination protocol, every drug interacts with its receptor or has the same receptor. So, knowing the mechanism of the combined drugs and their chemical features is essential to understanding the reason for the highest CSS and subsequently predicting the recommended combination therapy for clinical trials for every cancer type ^104^.

In Table 11, we added the mechanism of action for each drug and the type of cancer to the best scores dataset to figure out the relation of CSS, mechanism of each drug, and type of cancer. The top-scoring drug combination was reorganized based on the type of cancer tissue. Six types of cancer tissues arose and were studied. From Table 11, we observe the following.

- *Melanoma* topoisomerase II inhibitors or kinases inhibitors with mTOR inhibitors or inhibitors of phosphorylation at ser296-Chk1 (Kinases drugs). Inhibitors the function of Hsp90 as the anti-apoptotic drug is recommended. Drugs with top CSS values are Gemcitabine, MK-8776, BEZ-235, and Geldanamycin.
- *Ovarian cancer* the combination of drugs acting on kinases pathways e.g. CDK inhibitors, an inhibitor of FPTase, and GGPTase-I or inhibitors of the phosphorylation at ser296-Chk, Aurora-A kinases inhibitors or those acting on Alkylating agent, crosslink DNA. Drugs with top CSS values are L778123, MK-5108, Dinaciclib, MK-8776, or AZD1775.
- *Colorectal carcinoma* a combination of drugs acting on kinase pathways e.g. CDK inhibitors or inhibitors of the WEE1 activity and induces DNA damage or those acting as mTOR inhibitors. Drugs with top CSS values are AZD1775, BEZ-235, Bortezomib, or MK-8776.
- *Prostate cancer* kinases with WEE1 activity or induce DNA damage. Drugs with top CSS values are Bortezomib, Gemcitabine, BEZ-235, or AZD1775.
- *Breast cancer* Kinases with WEE1 activity and induces DNA damage. Using two drugs from the same mechanism as mTOR inhibitors is also recommended. Drugs with top CSS values are Gemcitabine, AZD1775, Bortezomib, or Geldanamycin.
- *Human lung cancer* Kinases or inhibitors of WEE1 activity induce DNA damage or kinases with mTOR inhibitor. HDAC inhibitor (Zolinza) is recommended. Drugs with top CSS values are AZD1775, MK-8776, Vinorelbine, Zolinza, or Gemcitabine.

By analyzing, the results based on the name of the drugs: Gemcitabine MK-8776, and AZD1775 were repeated among most of the cancer tissue.

In the quest to uncover potent combinations of drugs that can conquer diseases, a treasure trove of data awaits within Table 12. This comprehensive catalog, meticulously curated from diverse sources, holds the keys to unlocking drug synergy across a spectrum of illnesses. Each row within this table represents a unique study, a testament to the collective efforts of researchers around the globe. Together, they paint a tapestry of knowledge, weaving insights from cancer to SARS-CoV-2, malaria, and Ebola. The columns description contained the following attributes.

- STUDY NAME: The beacon that guides us to the source of knowledge, revealing the specific research endeavor.
- DISEASE: The battlefield where the fight unfolds, ranging from cancer's formidable frontlines to the emergent threats of viral foes.
- DATA SOURCE: The origin of the data, whether gleaned from published papers, curated databases like NCATS Tripod, or industry pioneers like AstraZeneca.
- PUBMED ID: A quick-link to delve deeper into the study's methodology and findings, allowing us to trace the footsteps of researchers.
- NUMBER OF DRUGS: The cast of characters, the arsenal of molecules wielded in the pursuit of synergistic magic.
- NUMBER OF BLOCKS: A glimpse into the complexity of the study, revealing the number of distinct combinations explored in this intricate dance.
- NUMBER OF CELL LINES: The battleground where the drugs are tested, the diverse landscapes of cells that bear witness to the power of synergy.
- NUMBER OF TISSUES: For studies venturing beyond the cellular level, this column reveals additional testing grounds, offering insights into drug synergy within the body's intricate tapestry.
- FULL DOSE–RESPONSE MATRIX SIZE: A window into the vastness of data collected, showcasing the scope of each investigation.

**Table 12:** Drug combination datasets used to confirm our results (https://drugcomb.org/help/#line12).

Study name	Disease	Data source	Pubmed id	Number of drugs	Number of blocks	Number of cell lines	Number of tissues	Full dose–response matrix size
ONEIL	Cancer	Publication	26983881	38	92,208	39	6	5 × 5
CLOUD	Cancer	Publication	28530711	283	40,160	1	1	2 × 2
ALMANAC	Cancer	Publication	28446463	103	311,604	60	9	4 × 4, 4 × 6
FORCINA	Cancer	Publication	28601558	1818	1818	1	1	2 × 2
NCATS_ATL	Cancer	NCATS Tripod		22	30	1	1	10 × 10
MATHEWS	Cancer	NCATS Tripod	24469833	477	1119	1	1	6 × 6, 10 × 10
NCATS_DIPG	Cancer	NCATS Tripod		2450	8854	2	2	6 × 6, 10 × 10
NCATS_ES(FAKI/AURKI)	Cancer	NCATS Tripod		1909	1910	1	1	6 × 6
NCATS_ES(NAMPT + PARP)	Cancer	NCATS Tripod		94	4628	4	3	6 × 6, 10 × 10
WILSON	Cancer	NCATS Tripod	30289729	31	764	2	1	6 × 6, 10 × 10
NCATS_HL	Cancer	NCATS Tripod		1910	2694	4	2	6 × 6, 10 × 10
YOHE	Cancer	NCATS Tripod	29973406	25	270	3	2	10 × 10
NCATS_2D_3D	Cancer	NCATS Tripod		5	70	2	2	10 × 10
PHELAN	Cancer	NCATS Tripod	29925955	16	62	1	1	10 × 10
NCATS_MDR_CS	Cancer	NCATS Tripod		18	68	2	1	10 × 10
CCLE	Cancer	PharmacoDB	22460905	24	11,670	503	24	6 × 1, 7 × 1, 8 × 1
CTRPV2	Cancer	PharmacoDB	26482930	544	395,263	887	24	8 × 1 ~ 29 × 1
FIMM	Cancer	PharmacoDB	24056683	52	2561	50	5	5 × 1
GCSI	Cancer	PharmacoDB	27193678	16	6455	409	23	8 × 1, 9 × 1
GDSC1	Cancer	PharmacoDB	23180760	250	225,480	1074	30	5 × 1, 9 × 1
GRAY	Cancer	PharmacoDB	24176112	89	9413	70	2	9 × 1
UHNBREAST	Cancer	PharmacoDB	26771497	4	52	15	1	9 × 1, 18 × 1
BEATAML	Cancer	Publication	30333627	122	59,348	528	1	7 × 1
FLOBAK	Cancer	Publication	31664030	19	9984	8	7	6 × 6
ASTRAZENECA	Cancer	AstraZeneca	31209238	116	20,482	153	10	6 × 6
FRIEDMAN	Cancer	Publication	26461489	108	208,008	36	1	3 × 3
SCHMIDT	Cancer	Publication	24101737	4	100	5	1	8 × 8
MILLER	Cancer	Publication	24065146	13	82	1	1	8 × 8
FRIEDMAN2	Cancer	Publication	28446504	76	28,500	10	1	3 × 3
TOURET	SARS-CoV-2	Publication	32753646	1516	1520	1	1	1 × 1
GORDON	SARS-CoV-2	Publication	32353859	75	290	1	1	5 × 1, 6 × 1, 7 × 1
ELLINGER	SARS-CoV-2	ChEMBL		5604	5632	1	1	1 × 1
MOTT	Malaria	NCATS Tripod	26403635	223	17,072	3	1	6 × 6, 10 × 10
NCATS_SARS-COV-2DPI	SARS-CoV-2	NCATS Tripod		56	206	1	1	6 × 6
BOBROWSKI	SARS-CoV-2	NCATS Tripod	32637956	34	262	1	1	6 × 6
DYALL	Ebola	NCATS Tripod	29939303	17	432	2	2	6 × 6
FALLAHI-SICHANI	Cancer	Publication	28069687	10	111	5	1	10 × 1, 20 × 1

The proposed model, meticulously trained to uncover patterns within this symphony of data, has achieved promising results in both regression and classification tasks across these diverse datasets. We proudly present only the best results in our evaluation, showcasing the potential of computational approaches to illuminate the path towards effective drug combinations. Table 12 beckons researchers and enthusiasts alike to explore its depths. Within its rows lies the promise of a future where drug synergy triumphs over disease, where the combined might of molecules paves the way towards a healthier world.

#### Drug descriptors

The molecular descriptors of each drug are responsible for the relevant activity^105^. These descriptors have been extensively demonstrated as a measure of structure. The selected drugs were represented as color atoms based on ROCS, as investigated in Table 13. ROCS is a feature and application in the Openeye scientific program (Academic License by Yaseen Elshaier 2021, https://www.eyesopen.com/). The characters include the following items:-

- *No acceptors* Several drug features can form HB as acceptors.
- *No donors* Several drug features can form HB as a donor.
- *No hydrophobe* Several drug features act as hydrophobe parts.
- *No of rings* number of rings inside the drug's chemical structure.
- *No of anion* number of anion groups inside the drug's chemical structure.
- *No cation* number of cation groups inside the drug's chemical structure.

**Table 13:** The number of color atoms for selected drugs calculated by ROCS.

Drug name	No of acceptors	No of donors	No of rings	No of hydrophobe	No of anion	No of cation
ZOLINZA	2	2	1	2	–	–
MK-5108	5	1	4	–	–	–
GEMCITABINE	6	4	2	–	–	
AZD1775	6	4	2	–	–	–
L778123	3	1	4	–	–	2
MK-8776	3	3	4	1	–	1
MK-8776	2	3	6	–	–	1
GELDANAMYCIN	8	3	2	–	–	
BORTEZOMIB	4	2	2	1	–	–
DINACICLIB	2	3	4	1	1	–
VINBLASTINE	6	5	8	2	–	1
VINORELBINE	5	4	9	2	–	1
BEZ-235	4	–	6	1	–	1

For selected drugs, the shape of the atoms and the corresponding color atoms determined by ROCS is shown in Table 13.

We found a combination of drugs complimentary by analyzing the results. Drugs with cationic or anionic features combined with drugs devoted from any cationic or anionic features. The number of acceptors, the number of donors, and the number of rings are very important. Total summation for the same type of descriptor shouldn’t be very high.

#### Cheminformatics study (ROCS analysis)

The ROCS assesses the three-dimensionality of medicines. It computed the shape and color of ligands in their binding proteins, which are crucial elements in determining commonalities between them. The ROCS program OpenEye scientific software displays shape and color attributes. The query molecules were chosen for their high degree of similarity [https://www.eyesopen.com/]. The database file was chosen as the Compounds library. Omega program reduced the amount of energy used by the database files. ROCS operates on a personal computer using the vROCS interface. vROCS was employed to run and analyze/visualize the results. The Vida application visualized the outcome. Compound conformers were rated based on their Gaussian overlap with the query, with Tanimoto Combo scores (shape + color) being the best scoring criteria. The compound with the highest score was the best matched with the query compound.

## Discussion, advantages, and limitation

This paper presents a machine-learning-driven approach to forecast effective drug synergy pairs for cancer treatment, encompassing multiple steps such as data collection, annotation, preprocessing, and model building. Notably, the study annotates drug combinations with generic names and mechanisms of action, contributing to a nuanced understanding of synergy behavior. Utilizing a mix of classification and regression models, the framework demonstrates its versatility. Integration of the ROCS adds a three-dimensional assessment of medicines, considering shape and color attributes. Furthermore, the analysis extends beyond drug combinations, classifying data by cancer tissue type and providing specific recommendations for different cancer types. Despite these strengths, the study relies on the ROCS program, introduces assumptions about drug-receptor interactions, and may oversimplify complex relationships between Combined Synergy Scores (CSS), drug mechanisms, and cancer types.

The study demonstrated several strengths in its approach to predicting synergistic drug combinations such as:

- It took a comprehensive, systematic view by incorporating multiple machine learning steps to build models. This enhances the robustness of the methodology.
- Annotating drug pairs with generic names and mechanisms of action provided more context around synergy behaviors.
- The versatility of both classification and regression models showcased the approach's flexibility in predicting synergy.
- Incorporating the ROCS program offered a three-dimensional perspective on drug structures and properties, providing shape- and color-based insights.
- Classifying data by cancer tissue type customized recommendations for different cancer types, considering their specificities.

The limitations of this work can be summarized as follows:

- Reliance on the ROCS program introduced potential issues from its own algorithms and information sources.
- Assuming all drugs interact through a single receptor oversimplified complex real-world interactions.
- While mechanisms were included, the completeness and standardization of this drug information could vary widely.
- Simplifying the relationship between CSS, drugs and cancers may have obscured intricate linkages.
- The model’s effectiveness depended heavily on the quality and representation of its underlying dataset.
- Seeing some drugs repeated across cancers raised questions about bias and generalizability.
- The lack of external validation with new data left applicability to other scenarios uncertain.

## Conclusion and future works

The proposed framework highlighted the importance of drug mechanisms in drug combination therapy decisions. This paper uses a classification model to classify three types of pharmacological combinations: synergism, additive, and antagonistic. This guarantees that the medication combination is effective on the designated cell. Furthermore, we applied machine learning algorithms to predict the drug combination sensitivity score to enhance the results with real data. The experimental results pass different stages to achieve the required classification and prediction tasks. Starting with the preprocessing stage, the normalization, outlier removable, and data balancing were performed. Afterward, the enrolled data are ready for classification and prediction using two intervals applied to the O'Neil drug combination data. With machine learning techniques, we have concluded that drug combinations significantly impact the physician's decision-making in choosing the best method for inclusion. In the future, we plan to use AI applications to predict the different types of drug combination therapy for alleviating a series of diseases.

As for the future works, there are several futures that will shape how we predict optimal drug combinations in the years ahead. The future research focused more on integrating different types of biological data, like genetics, gene expression, proteins, and metabolites. Looking at all these “omics” together can give us a more complete picture of how drugs work together and interact at the molecular level, helping identify the best combinations. Artificial intelligence, especially deep learning, will also play a bigger role. These advanced algorithms excel at finding complex patterns in data, which is perfect for capturing the nonlinear relationships inside our bodies. This should lead to more accurate predictions of synergistic drug effects.

## Data Availability

The dataset used in this study is public and all test data are available at this portal (https://drugcomb.fimm.fi). DrugComb is an open-access, community-driven data portal where the results of drug combination screening studies for a large variety of cancer cell lines are accumulated, standardized, and harmonized. An actively expanding array of data visualization and computational tools is provided to analyze drug combination data. All the data and informatics tools are made freely available to a broader community of cancer researchers.

## Abbreviations

CSS: Combination sensitivity scores
DNA: Deoxyribonucleic acid
FDA: Food and Drug Administration
GCN: Graph convolutional network
HDAC: Histone deacetylase
IQR: Inter-quartile range
KNN: K-nearest neighbors
LR: Logistic regression
ML: Machine learning
MAE: Mean absolute error
MSE: Mean squared error
NB: Naive Bayes
RF: Random forest
ROCS: Rapid overlay chemical similarity
R^2^: (R-squared) coefficient of determination
